# Selective Scandium (Sc) Extraction from Bauxite Residue (Red Mud) Obtained by Alkali Fusion-Leaching Method

**DOI:** 10.3390/ma15020433

**Published:** 2022-01-07

**Authors:** Andrei Shoppert, Irina Loginova, Julia Napol’skikh, Aleksey Kyrchikov, Leonid Chaikin, Denis Rogozhnikov, Dmitry Valeev

**Affiliations:** 1 Laboratory of Advanced Technologies in Non-Ferrous and Ferrous Metals Raw Materials Processing, Ural Federal University, 620002 Yekaterinburg, Russia; darogozhnikov@yandex.ru; 2 Department of Non-Ferrous Metals Metallurgy, Ural Federal University, 620002 Yekaterinburg, Russia; i.v.loginova@urfu.ru (I.L.); anapolskikh512@gmail.com (J.N.); akirchikov@yandex.ru (A.K.); l.i.chaikin@urfu.ru (L.C.); 3 Laboratory of Sorption Methods, Vernadsky Institute of Geochemistry and Analytical Chemistry, The Russian Academy of Sciences, 119991 Moscow, Russia; dmvaleev@yandex.ru

**Keywords:** waste recycling, rare earth elements, acid leaching, kinetics, shrinking core model

## Abstract

Bauxite residue, known as “red mud,” is a potential raw material for extracting rare-earth elements (REEs). The main REEs (Sc, Y, La, Ce, Nd, Nb, and Sm) from the raw bauxite are concentrated in RM after the Bayer leaching process. The earlier worldwide studies were focused on the scandium (Sc) extraction from RM by concentrated acids to enhance the extraction degree. This leads to the dissolution of major oxides (Fe_2_O_3_ and Al_2_O_3_) from RM. This article studies the possibility of selective Sc extraction from alkali fusion red mud (RMF) by diluted nitric acid (HNO_3_) leaching at pH ≥ 2 to prevent co-dissolution of Fe_2_O_3_. RMF samples were analyzed by X-ray fluorescence spectrometry (XRF), X-ray diffraction (XRD), electron probe microanalysis (EPMA), and inductively coupled plasma mass spectrometry (ICP-MS). It was revealed that Sc concentration in RMF can reach up to 140–150 mg kg^−1^. Sc extraction was 71.2% at RMF leaching by HNO_3_ at pH 2 and 80 °C during 90 min. The leaching solution contained 8 mg L^−1^ Sc and a high amount of other REEs in the presence of relatively low concentrations of impurity elements such as Fe, Al, Ti, Ca, etc. The kinetic analysis of experimental data by the shrinking core model showed that Sc leaching process is limited by the interfacial diffusion and the diffusion through the product layer. The apparent activation energy (E_a_) was 19.5 kJ/mol. The linear dependence of Sc extraction on magnesium (Mg) extraction was revealed. According to EPMA of RMF, Sc is associated with iron minerals rather than Mg. This allows us to conclude that Mg acts as a leaching agent for the extraction of Sc presented in the RMF in an ion-exchangeable phase.

## 1. Introduction

Red mud (RM) is a solid waste generated by alkali (NaOH) leaching of bauxite by the Bayer process for alumina production [1]. More than one ton of RM is produced for one ton of alumina (Al_2_O_3_), and over 150 Mt of RM is annually stored in landfills and sea [2]. The RM utilization degree is extremely low due to the high Na, Al, Si, and Ca content because these elements increase the consumption of reagents during subsequent RM processing to extract valuable components [3]. The high content of toxic elements (Na, Cd, Ni, As, Sb, Pb, and Cr) and small particle size of RM (<10 µm) lead to contamination of soil, air, and water around the landfills [4,5].

In addition to RM obtained by the Bayer process, there is a sintering RM. It is obtained during sintering high-silica bauxite with soda (Na_2_CO_3_) and limestone (CaCO_3_) and further water leaching [6]. The sintering RM contains less caustic alkali (not more than 5 wt%) [7], but its CaO content is much higher (up to 42 wt%) [8]. This type of RM can generally be utilized as a construction material due to low iron oxide (Fe_2_O_3_) content and a high content of CaO and SiO_2_ [9]. Alumina refineries that use combined methods—Bayer and sintering (Urals Alumina Refinery, Kamensk-Uralsky, Russia and Bogoslovsk Alumina Refinery, Krasnoturinsk, Russia)—usually store both types of waste on one landfill [10,11].

Despite the method of bauxite processing, RM contains a high number of valuable components, especially rare earth elements (REEs) that are widely used in high-tech materials production [12,13,14]. REEs do not dissolve during bauxite alkali leaching and concentrate in the solid residue with an enrichment factor of about two [15,16]. Main studies on the REEs extraction from RM are devoted to scandium (Sc) extraction [3,6,9,15].

The Sc demand annually increases as it is used in lasers glass, catalysts, electronic components, halide lamps, fuel cells, and non-ferrous metallurgy [17]. The most relevant application is the production of Al-Sc alloys with high strength and low weight [18]. The Sc content in different types of RM (more than 100 mg kg^−1^) is higher than the average abundance in the Earth’s crust (22 mg kg^−1^) [19]. The vast amount of accumulated RM (more than 4 Bt) [20] can be used as raw material for the Sc extraction.

One of the more promising and eco-friendly methods for the Sc extraction is sodium bicarbonate (NaHCO_3_) leaching [21,22] and carbonization [23]. United Company RUSAL launched a pilot plant by NaHCO_3_ leaching in 2014 [24]. Sc extraction degree by this method does not exceed 30–40%, even at the application of the sorption in pulp process [25].

The most effective methods for Sc extraction are RM leaching by dilute inorganic acids [26,27,28,29]. Rivera et al. found that for a high Sc extraction degree, complete iron oxide leaching is also necessary [30]. Sc is primarily associated with iron minerals in RM in the form of ions adsorbed on the surface or replacing other ions in the solid matrix [31]. High iron content in the acid solution complicates the subsequent Sc extraction since these metals have similar physical and chemical properties [17]. It was shown [32] that acid leaching was also the most effective method for REEs extraction from other raw materials, i.e., coal related materials, and it was shown that alkaline and thermal pretreatment could considerably enhance leaching efficiency.

Ochsenkuehn-Petropoulou et al. [27,33] showed that the Sc extraction from the sintering RM could be carried out at a pH > 1.5. However, the extraction degree of Sc was lower than 80% and 40% at a solid-to-liquid ratio of 2% and 30%, respectively. This reduces the Fe extraction; however, acid consumption is significant due to reaction with CaO. Anawati et al. [34] found that it is possible to increase Sc extraction by fusing RM with sulfuric acid or alkali. During the RM fusing, iron minerals undergo phase transformation that can lead to the liberation of REEs from the solid matrix of the minerals. Therefore, obtaining RM by fusing may provide an alternative for the selective extraction of REEs without Fe, Al, and Ca co-dissolution.

This research studied the selective Sc extraction from RM obtained by fusing bauxite with NaOH to optimize the leaching process at pH > 2. A diluted nitric acid (HNO_3_) was chosen as the leaching agent since Reid et al. [35] showed that the Sc extraction degree, in this case, was higher by ~10% in comparison with H_2_SO_4_ leaching at the same concentration and temperature. The influence of pH, the liquid to solid ratio (L:S), temperature, and leaching time on Sc extraction have been investigated. The mechanism and kinetics of leaching have been studied using the shrinking core model.

## 2. Materials and Methods

### 2.1. Analysis

The phase composition of RM was detected by powder X-ray diffraction spectrometry (XRD) via Rigaku D/MAX-2200 diffractometer (Rigaku Co., Tokyo, Japan) equipped a Cu-Kα radiator (λ = 1.541841 Å). Chemical analysis of the major elements in RM was performed by powder X-ray fluorescence spectrometry (XRF) with an Axios MAX spectrometer (Malvern Panalytical Ltd., Almelo, The Netherlands). Chemical analysis of the minor elements was analyzed by inductively coupled plasma mass spectrometry (ICP-MS) via NexION 300S instrument (PerkinElmer, Waltham, MA, USA). The morphology of RM particles was evaluated using the scanning electron microscopy (SEM) via JSM-6390 instrument (JEOL, Tokyo, Japan). The mapping of the trace and major elements in RM samples was performed using electron probe micro-analysis (EPMA) via a Cameca SX 100 microanalyzer (CAMECA Instruments, Inc., Madison, WI, USA) with an energy-dispersive X-ray spectroscopy analysis (EDS) module XFlash 6 (Bruker Nano GmbH, Berlin, Germany). The loss on ignition (LOI) was determined by heating RM samples from 50 to 1000 °C at the rate of 20 °C/min using a Diamond TG/DTA instrument (PerkinElmer, Waltham, MA, USA).

### 2.2. Materials

**Figure 1:**
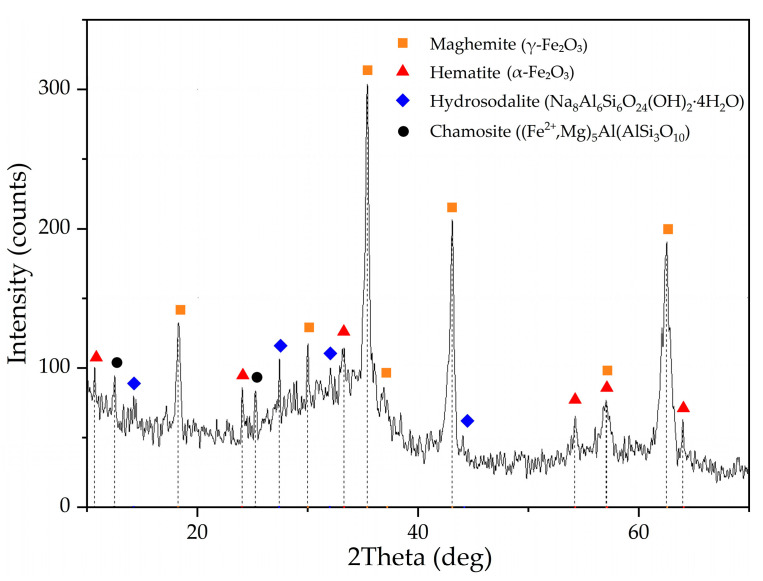
XRD pattern of the initial red mud obtained by alkali fusion-leaching of bauxite (RMF).

RM samples were obtained by alkali fusion-leaching method from bauxite of Middle-Timan deposit (Ukhta, Russia). The chemical composition and properties of the raw bauxite and the red mud obtained by the alkali fusion-water leaching method from this bauxite (RMF) and the mechanism of the process were presented in our previous article [36]. Alkali fusion conditions used to obtain RMF in this research were as follows: T = 300 °C, fusion time 120 min, bauxite/NaOH weight ratio 1:1. The fusing product was leached by water at T = 80 °C, L:S ratio = 3.5, and leaching time 30 min. RMF was filtered, washed by distilled water, and dried at T = 110 °C. RMF chemical composition is presented in Table 1. RMF contains a high amount of Fe and a low amount of Na, Al, and Si compared to Bayer RM [36]. XRD of RMF is shown in Figure 1. Almost all iron in RMF is presented as maghemite (γ-Fe_2_O_3_). The latter is formed by hydrolysis of sodium ferrite (NaFeO_2_), which is the main iron phase of the fusing product. Some amount of hematite (Fe_2_O_3_) and chamosite ([Fe^2+^,Mg]_5_Al[AlSi_3_O_10_]), the main iron phases of raw bauxite, still can be seen in RMF obtained by the fusion-leaching process.

**Figure 2:**
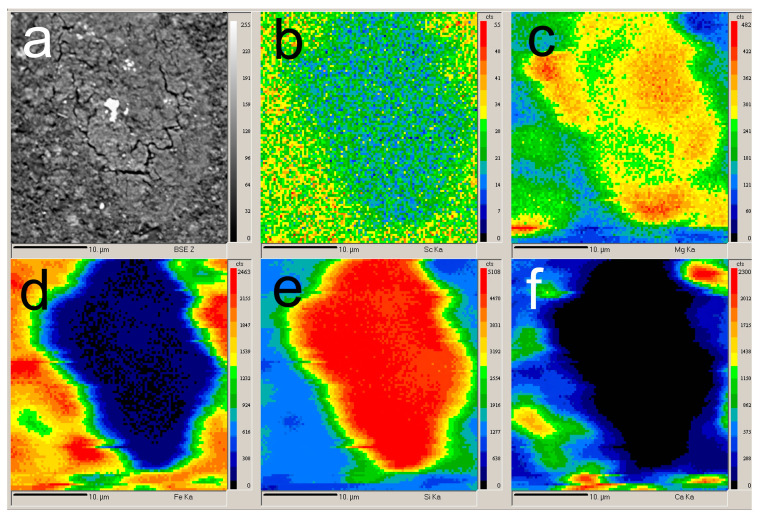
EPMA of the initial red mud obtained by alkali fusion-leaching of bauxite (RMF): (**a**) BSE image of the RMF surface; (**b**) mapping of the Sc distribution; (**c**) mapping of the Mg distribution; (**d**) mapping of the Fe distribution; (**e**) mapping of the Si distribution; and (**f**) mapping of the Ca distribution.

**Figure 3:**
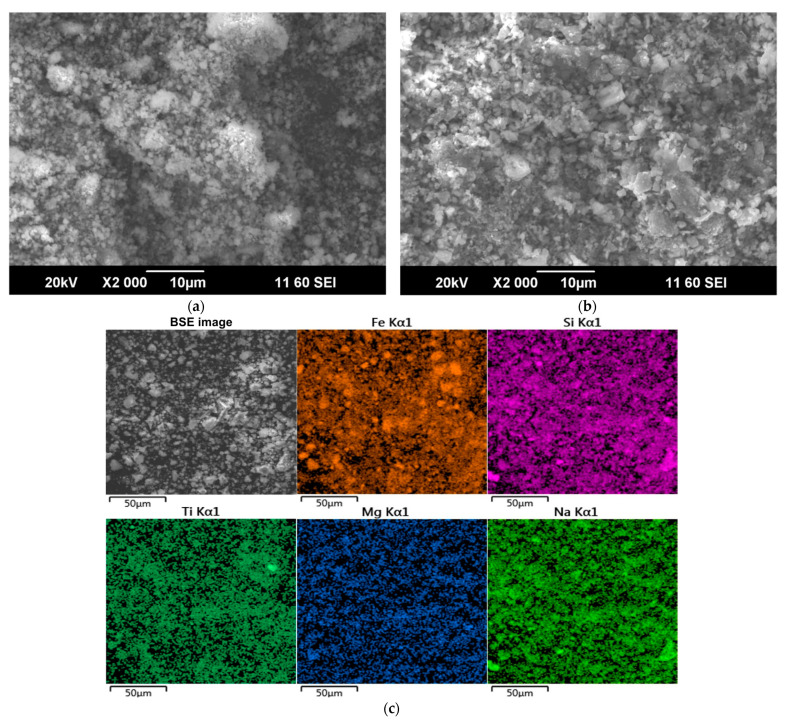
SEM images of: (**a**) the initial red mud obtained by alkali fusion-leaching of bauxite (RMF); (**b**) the Bayer red mud obtained from Middle Timan bauxite treatment on Urals Alumina Refinery; and (**c**) SEM image and EDS elemental mapping of RMF.

According to previous research [31,37,38,39], Sc is commonly adsorbed on Fe_2_O_3_ and in channels of the aluminosilicates that is formed during desilication of pregnant solution [25]. During the alkali fusion-leaching method, hematite and chamosite are transformed into maghemite. Desilication product (DSP), in this case, is hydrosodalite (Na_8_Al_6_Si_6_O_24_[OH]_2_·4H_2_O). DSP amount is very low, as can be seen from Table 1 and Figure 1. Therefore, Sc could be liberated from the solid matrix of bauxite minerals, and its amount adsorbed in DSP is much less than in typical RM with a high concentration of DSP. However, scandium could be adsorbed on the Fe_2_O_3_ that formed after the alkali fusion-leaching method. This fact could lead to an increased REEs extraction compared to Bayer RM treatment. The EPMA analysis showed the association of Sc with the main components (Fe, Si, Ca, and Mg) in RMF (Figure 2). The mapping analysis confirms that Sc is associated more with Fe_2_O_3_ than with other minerals. Figure 3 shows the SEM images of RMF and Bayer RM, obtained from Middle Timan bauxite treatment on Urals Alumina Refinery. It can be seen in Figure 3 that RMF consists of small particles of approximately the same size of several microns or less. The Bayer RM particles are larger; this RM consists of particles of different sizes (0.2–5 μm). The EDX mapping of RMF (Figure 3c) shows that the distribution of elements is uniform. However, particles with high iron concentrations are more common. The association of Na with Si indicates that Na in the RMF is presented in hydrosodalite; Mg distribution is homogenous.

### 2.3. Experiments

The leaching of RMF in diluted nitric acid (HNO_3_) was carried out in a 0.5 L thermostatic reactor “Lenz Minni” (Lenz Laborglas GmbH & Co. KG, Wertheim, Germany) fitted with an overhead stirrer. The 10, 15, or 20 g of RMF was mixed into the 180 mL of distilled water, then 20 mL of HNO_3_ solution with the predetermined concentration was added, according to the RMF sample weight and desired pH. The pH of the solution was kept constant by the addition of 1M HNO_3_ solution using Automatic titrator ATP-02 (JSC Akvilon, Podolsk, Russia).

After leaching, the solid residue was separated from the acid solution by filtration on a Buchner funnel. The Sc, Fe, Al, and Mg content in the solution were measured using ICP-OES. After washing and drying of the solid residue for 8 h at T = 110 °C, it was used for SEM and XRF analysis.

The elements extraction degree was calculated by the following Equation (1):

η_Me_ = C_Me_ × V × 100%/(m × X_Me_), (1)

where C_Me_ is the concentration of a metal in the solution (mg L^−1^), V is the volume of the solution (L), m is the mass of RMF (kg), X_Me_ is a content of a metal in RMF (mg kg^−1^).

The Eh–pH diagrams were calculated using the HSC Chemistry Software v. 9.9 (Metso Outotec Finland Oy, Tampere, Finland).

## 3. Results and Discussion

### 3.1. Effect of Leaching Conditions on the Sc Selective Leaching from RMF

**Figure 4:**
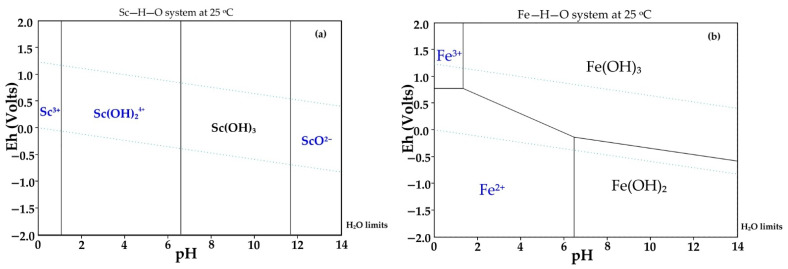
Thermodynamic modeling of: (**a**) the system Sc–H–O at 25 °C; and (**b**) the system Fe–H–O.

Selective Sc leaching was achieved due to the Sc and Fe solubility difference at pH > 2. Thermodynamics in the system Fe–H–O and Sc–H–O at 25 °C and different pH levels are shown in Figure 4. At pH 1.2 to 3 and high Eh (>0.5), Fe(OH)_3_ begins to precipitate. Unlike Fe, Sc begins to precipitate only at pH 6.5. In addition, the kinetics of Fe and Sc minerals leaching should be considered. Rivera et al. [19] showed that Sc extraction from industrial bauxite residue is very low at pH > 2.5. Thus, it is necessary to use concentrated acid for complete Sc leaching. The use of strong acid leads to high co-dissolution of iron.

**Figure 5:**
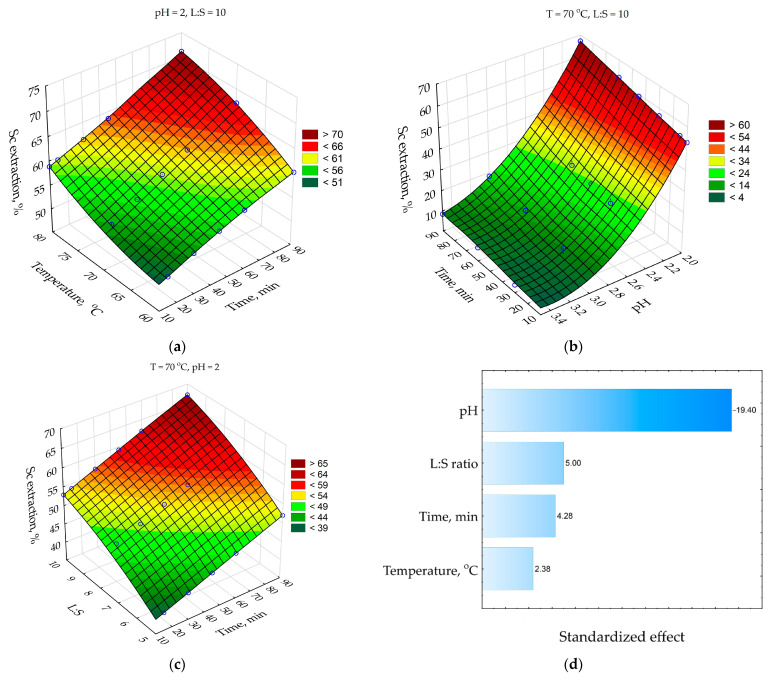
Response surfaces for: (**a**) effect of time and temperature on the Sc extraction; (**b**) effect of time and pH on the Sc extraction; (**c**) effect of time and L:S ratio on the Sc extraction; and (**d**) Pareto chart. Blue points are the experimental data.

The optimal pH range was defined as 2–3.5 for a higher Sc extraction selectivity and leaching rate. The effect of temperature (T, °C), leaching time (τ, min), pH, and liquid to solid ratio (L:S) on the Sc extraction with HNO_3_ from RMF is shown in Figure 5. A Pareto chart was constructed to estimate the standardized effect of each parameter on Sc extraction (Figure 5d).

As Figure 5a shows, the Sc extraction increases by 10–15% with an increase in time from 10 to 90 min at all temperatures. An increase in temperature from 60 to 80 °C after 90 min of leaching leads to an increase in Sc extraction by 12%. The low effect of leaching time and temperature at later stages of leaching (when >50% of Sc was extracted) could be a sign of the diffusion limitation (reagent diffusion to the surface of the reaction through the product layer or the liquid film).

The pH effect on the Sc extraction is shown in Figure 5b. At high pH = 3–3.5, the Sc extraction was lower than 20% despite leaching time, but it increases when the pH level reduces to 2–2.5. The decrease in pH from 3.5 to 2 increases Sc extraction by about 60% after 90 min of leaching at T = 70 °C. Therefore, maintenance of pH is significant for a high Sc extraction from RMF. An increase in the L:S ratio from 5 to 10 also increases Sc extraction by 18% after 90 min of leaching at T = 70 °C (Figure 5c). It can be related to the high amount of free acid that reacts with Sc compounds. The Pareto chart (Figure 5d), where the standardized effect of each parameter is given, confirms the above observations. The pH effect is four times higher than the L:S ratio and leaching time effects. The standardized effect of temperature is about two times lower than leaching time.

### 3.2. Study of the Kinetics and Mechanism of Sc Leaching from RMF

The higher effect of the L:S ratio in comparison with temperature on the Sc extraction indicates that diffusion is the limiting stage of the leaching process because, at the high L:S ratio, the amount of free leaching reagent is higher. However, after 10 min of leaching, the Sc extraction degree is higher than 50% at pH 2. Therefore, at the initial time, the surface chemical reaction could be the limiting stage, and to confirm it, the kinetics was evaluated at the first 5 min of leaching.

The shrinking core model (SCM) is usually used to characterize the kinetics of the heterogenic leaching process [40]. The SCM assumes that the leaching rate is controlled by surface chemical reaction, diffusion through the liquid film, or diffusion through the porous solid product. The unreacted core of the particle covered by this product shrinks to the center during the reaction. Several kinetic equations were proposed to determine the limiting stage of the process (Equations (2)–(4), Table 2).

**Figure 6:**
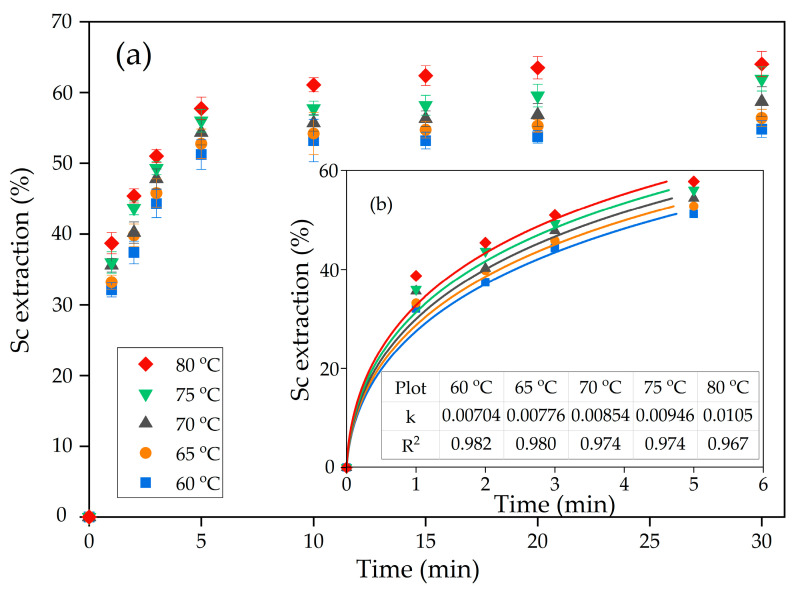
Dependence of: (**a**) the degree of scandium extraction on temperature at L:S ratio 10:1 and pH 2; and (**b**) fitting of experimental data of leaching during the first 5 min to the new shrinking core model.

The least-square graphical method (Figure 6) was used to fit experimental data to each equation from Table 2. The results of fitting for Equation (4) are shown in Figure 6b.

1 − 3(1 − X)^2/3^ + 2(1 − X) = k_1_τ * (2)

1 − (1 − X)^1/3^ = k_2_τ (3)

1/3ln(1 − X) + [(1 − X)^−1/3^ − 1] = k_3_τ (4)

where k_i_ is the corresponding rate constant.

As Figure 6 and Table 2 reveal, the surface reaction equation suits less for the characterization of Sc leaching kinetics, as R^2^ is <0.8 for all temperatures. The new shrinking core model (Equation (4), Table 2) is more suitable for fitting the experimental data. This model assumes that interfacial diffusion and diffusion through the product layer are the limiting stages of the leaching process [41].

**Figure 7:**
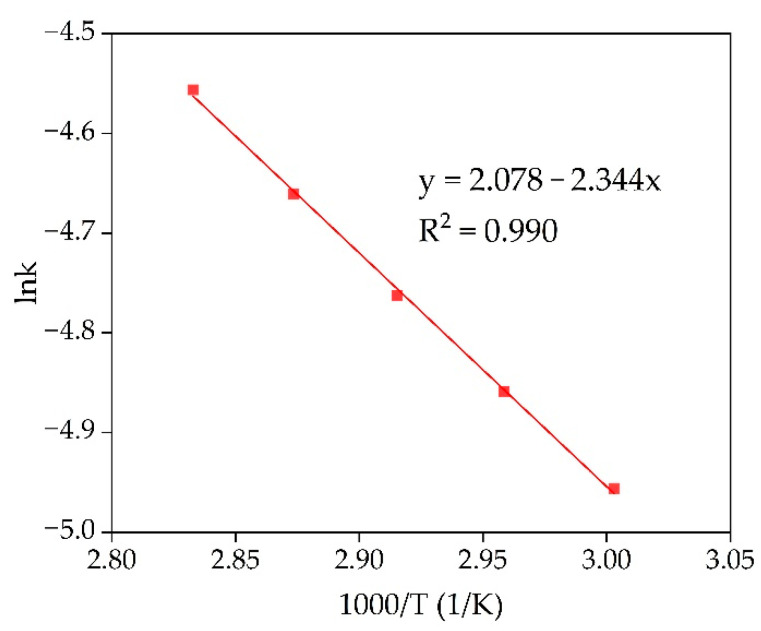
Dependence of lnK on 1000/T (Arrhenius plot) for Sc extraction from RMF in temperature range from 60 °C to 80 °C.

The Arrhenius plot was created in lnK-1000/T coordinates to characterize further the limiting stage of the leaching process (Figure 7). It applied the rate constants showed in Figure 6b. The linear fitting was performed to obtain equation y = ax + b, where a is the slope of the curve used to find the apparent activation energy using Equation (4).

The obtained value of the apparent activation energy is 19.5 kJ/mol. The shrinking core model and the value of the activation energy confirm that diffusion of the reagent to the reaction surface is the limiting stage of the Sc leaching process. The product layer that can prevent reagent diffusion to the reaction surface can be hematite or chamosite that did not react with NaOH (according to Figure 1) and remains inert in a slightly acidic condition (pH > 2). Although very fine maghemite particles (less than 1 µm, Figure 3a), with a high specific surface area and porosity [36], could be the product layer that prevents diffusion as they are almost inert at such pH values.

**Figure 8:**
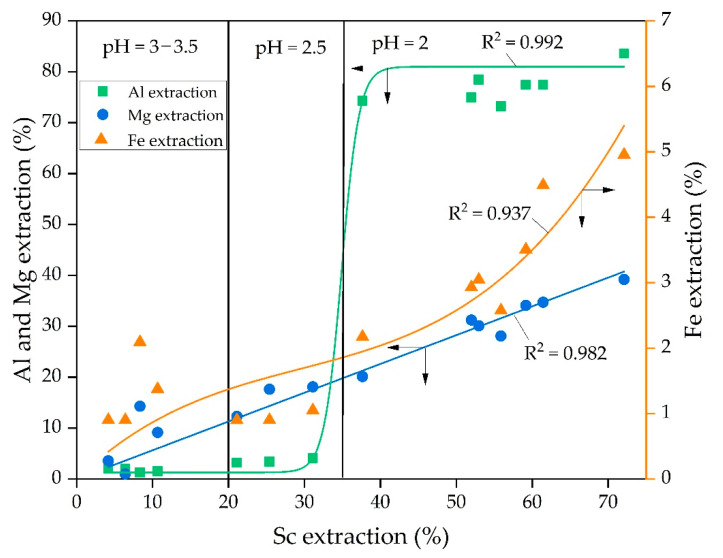
Dependence of the Al, Mg, and Fe extraction on Sc extraction and the fitting of the experimental data obtained at different pH value.

The experiments concerning the dependence of Al, Mg, and Fe extraction on Sc extraction (Figure 8) were performed to study the leaching process mechanism. The experimental data were subjected to the linear and non-linear fitting using 3rd order polynomic and Boltzmann function [42]. The Boltzmann function gives a sigmoidal curve and is well suited to characterize processes with a great increase in values. According to Figure 8, the Boltzmann function y = 81 + (1.3 − 81.0)/(1 + exp(x − 34.9)/1.2) suits better (R^2^ = 0.992) for a description of the dependence of Al extraction on Sc extraction. This can be explained by the fact that almost all Al in RMF is contained in the hydrosodalite (Figure 1). Hydrosodalite begins to dissolve at pH > 2; thus, a great increase in the Al extraction degree is observed. According to Figure 2, the Sc amount associated with aluminosilicate is low. Petrakova et al. [25] showed that Sc associated with the DSP accounts for more than 30% of Sc in Bayer RM. Therefore, the increase in aluminosilicate dissolution could raise the Sc extraction degree.

According to the EPMA analysis of RMF (Figure 2) and our previous research [43], it was found that the association of Sc with iron-containing minerals is high. After alkali-fusing of bauxite followed by water leaching, the hematite matrix was destroyed, and the amount of the hydrosodalite was very low (Table 1, Figure 1). Also, maghemite that formed after water leaching has a high specific surface area value (>50 m^2^ g^−1^ vs. 10–20 m^2^ g^−1^ for industrial RM [36]), so Sc is predominantly adsorbed on its surface. Therefore, the leaching of REEs from this type of RM should be more efficient. However, at pH > 2, when the Sc extraction was lower than 50%, Fe extraction was less than 3%, but when Sc extraction increases to 60–70%, the Fe extraction increases by a factor of two. It could be explained as follows. Some hematite and chamosite remain in the solid residue (Figure 1), so its solid matrix should be destroyed at lower pH for Sc extraction. This fact leads to an increase in the Fe and Sc extraction. Thus, the polynomic curve (y = 0.111x − 0.003x^2^ + 3.117 × 10^−5^x^3^) suits the best (R^2^ = 0.937) for a description of the dependence of Fe extraction on Sc extraction, as shown in Figure 8. The microwave-assisted alkali fusion [44] and the fusion at higher temperatures could enhance Sc extraction from unreacted hematite and chamosite by obtaining a homogeneous product where all the iron minerals are converted into a new phase with the release of REEs from their solid matrix.

**Figure 9:**
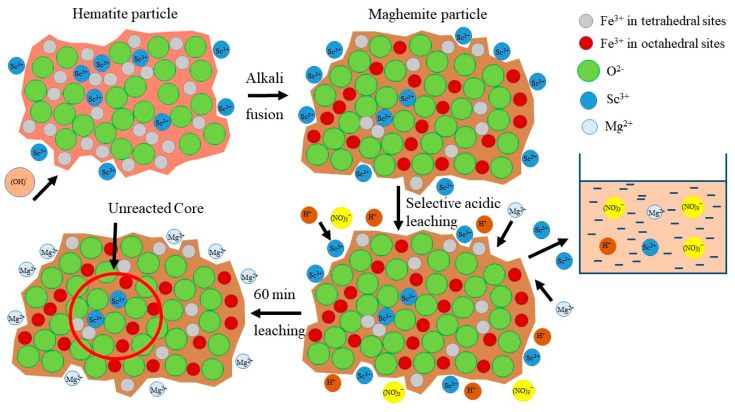
The mechanism of selective Sc leaching from RMF.

The dependence of Mg extraction on Sc extraction was almost linear (y = 0.565x, with R^2^ = 0.982). It means that the Sc extraction is associated with the Mg extraction at all pH values. However, according to EPMA analysis (Figure 2), the Sc association with Mg in RMF is low. He Q. et al. [45] and Xiao Y. et al. [46,47] showed that the ion-exchangeable phase of REEs could be easily extracted by MgSO_4_ solutions. Furthermore, it was revealed that Sc leaching could be performed even at pH 4 and higher using MgSO_4_ solution with the concentration 12–36 g L^−1^ that will be shown in our future article. Therefore, it can be assumed that a high amount of Sc, after the alkali fusion-leaching method, is presented in RMF in the ion-exchangeable phase. At the first step, Mg is extracted into the solution in the cationic form, then Mg^2+^ begins to act as a leaching agent. Thus, the linear dependence of Sc extraction on Mg extraction can be explained by this fact. Although Mg content in RMF was ~0.8 wt% (Table 1), it is more than 40 times higher than Sc content. The Mg extraction during the leaching process is ~40%. However, this content of Mg in the acid solution is enough to leach more than 70 wt% of Sc. The mechanism of Sc leaching from RMF is proposed in Figure 9.

The Fe_2_O_3_ content of the solid residue after Sc extraction was >76 wt% (Table 3). The Na_2_O content was lower than 1 wt%. The high content of maghemite nanoparticles with high specific surface area (>50 m^2^ g^−1^) and a low amount of other impurities (Table 3) determine its possible adsorption, ions-exchange, catalysts, and magnetism functions, as was shown in our previous article [36].

After RMF leaching at pH 2, T = 70 °C, L:S = 10 and τ = 30 min and removal of the insoluble residue by filtration, the leaching solution contained 8 mg L^−1^ Sc and high amount of other REEs in the presence of relatively low concentrations of impurity elements such as Fe, Al, Ti, Ca, etc. (Table 4). The low extraction efficiency of Ce, Nd, and Th can be explained by the formation of insoluble compounds at high pH and Eh values, as was shown by Lin P. et al. [48]. Sorption or extraction methods can be used to recover REEs from the resulting solution to increase the pregnant solution’s concentration before REEs precipitation [28].

## 4. Conclusions

This study presented the possibility of selective Sc leaching from the red mud obtained by the alkali fusion-leaching method using dilute nitric acid (pH 2–3.5). Compared with the industrial Bayer RM, RMF is a more promising source for REEs due to its high REEs content and low concentrations of Na, Ca, Al, and other undesirable elements that elevate acid consumption. Furthermore, the use of the fusing process leads to iron minerals phase transformation and the liberation of REEs from its solid matrix that enhance extraction efficiency of REEs.

The pH has a significant effect on the Sc extraction degree. With a decrease in pH from 3.5 to 2, the Sc extraction after 90 min of leaching at T = 70 °C increases from 10 wt% to 68 wt%; iron extraction increases from 1.3 wt% to almost 5 wt%. A low pH value is necessary for Sc leaching from the solid matrix of maghemite and iron minerals that were not destroyed at the alkali fusion of the raw bauxite. The temperature hasn’t such a significant effect on Sc extraction. A total of 71.2 wt% of Sc was extracted at pH = 2, T = 80 °C, L:S ratio 10, and leaching time 90 min. Decreasing leaching time from 90 min to 30 min at pH = 2, T = 70 °C, L:S ratio 10 decreases Sc extraction from 68 % to 57.5 %. However, the Fe extraction at these conditions was lower than 3 wt%.

Using an EPMA and XRD analysis, and the shrinking core model for kinetics and leaching mechanism investigation, the following main conclusions were obtained:

1. The results of the kinetics analysis show that the Sc leaching process is limited by the interfacial diffusion and the diffusion through the product layer, which could be formed by iron minerals (maghemite and hematite). The apparent activation energy Ea was 19.5 kJ/mol;
2. The linear dependence of Sc extraction on Mg extraction was revealed. According to EPMA analysis, Sc in the raw RM is associated with Fe rather than Mg. This allows us to conclude that Mg acts as a leaching agent for the extraction of Sc presented in the RMF in an ion-exchangeable phase;
3. A low pH value enhances the Mg leaching, which also increases the extraction of scandium.

The leaching solution contained more than 8 mg L^−1^ Sc and a high amount of other REEs. However, Ce, Nd, and Nb extraction were low, which could be attributed to the formation of insoluble compounds at high pH and Eh values. Due to its high specific area (>50 m^2^ g^−1^), high iron oxide content (>75 wt%), and magnetic properties, the maghemite containing solid residue obtained after Sc leaching can be used as a functionalized material.

## Figures and Tables

**Table 1:** Chemical composition of the red mud obtained by alkali fusion-leaching method (RMF).

**Major Components, wt%**											
Fe_2_O_3_	TiO_2_	SiO_2_	Al_2_O_3_	MnO	Na_2_O		MgO	CO_2_	CaO	ZrO_2_	LOI
65.50	7.79	6.02	5.04	1.29	0.78		0.75	0.65	0.39	0.26	11.52
**Minor Components, mg kg^−1^**											
CeO_2_		La_2_O_3_		Nd_2_O_3_		Nb_2_O_5_		Sc_2_O_3_		Y_2_O_3_	
862		365		322		241		212		196	

**Table 2:** SCM equation fitting for Sc extraction from RMF.

Equation No.	Fitting Results (R^2^)				
	60 °C	65 °C	70 °C	75 °C	80 °C
(2)	0.957	0.950	0.940	0.935	0.921
(3)	0.733	0.724	0.712	0.706	0.687
(4)	0.982	0.980	0.974	0.974	0.967

**Table 3:** Chemical composition of the red mud after selective REE leaching at pH 2, T = 70 °C, L:S = 10 and τ = 30 min.

**Major Components, wt%**											
Fe_2_O_3_	TiO_2_	SiO_2_	Al_2_O_3_	MnO	Na_2_O		MgO	CO_2_	CaO	ZrO_2_	LOI
76.27	8.87	6.80	1.41	1.48	0.93		0.66	1.05	0.23	0.25	1.59
**Minor Components, mg kg^−1^**											
CeO_2_		La_2_O_3_		Nd_2_O_3_		Nb_2_O_5_		Sc_2_O_3_		Y_2_O_3_	
904		156		76		201		110		20	

**Table 4:** The extraction efficiency and selected elements composition of the solution obtained after the RMF leaching at pH 2, T = 70 °C, L:S = 10 and τ = 30 min.

Element	Fe	Al	Ti	Ca	Mg	Na	Mn	Zr	La	Y	Nd	Ce	Sc	Nb	Th
Extraction, wt%	2.9	77.1	6.6	53.2	27.3	18.0	6.1	22.8	65.0	91.6	43.2	14.0	57.5	34.7	19.7
Concentration, mg L^−1^	1330	2050	394	191	157	132	78	57	26	18	15	13	8	7	3

## Data Availability

Data sharing is not applicable.
